# Two Arabidopsis late pollen transcripts are detected in cytoplasmic granules

**DOI:** 10.1002/pld3.12

**Published:** 2017-10-16

**Authors:** María R. Scarpin, Lorena Sigaut, Silvio G. Temprana, Graciela L. Boccaccio, Lía I. Pietrasanta, Jorge P. Muschietti

**Affiliations:** ^1^ Instituto de Ingeniería Genética y Biología Molecular “Dr. Héctor N. Torres” (INGEBI‐CONICET) Buenos Aires Argentina; ^2^ Instituto de Física de Buenos Aires (IFIBA‐CONICET) Departamento de Física Facultad de Ciencias Exactas y Naturales Universidad de Buenos Aires Ciudad Universitaria Buenos Aires Argentina; ^3^ Fundación Instituto Leloir IIBBA‐CONICET Facultad de Ciencias Exactas y Naturales Departamento de Fisiología y Biología Molecular y Celular Universidad de Buenos Aires Ciudad Universitaria Buenos Aires Argentina; ^4^ Centro de Microscopías Avanzadas Facultad de Ciencias Exactas y Naturales Universidad de Buenos Aires Ciudad Universitaria Buenos Aires Argentina; ^5^ Departamento de Biodiversidad y Biología Experimental Facultad de Ciencias Exactas y Naturales Universidad de Buenos Aires Ciudad Universitaria Buenos Aires Argentina

**Keywords:** MATLAB, MS2, pollen, processing body, translational regulation

## Abstract

Many of mRNAs synthesized during pollen development are translated after germination, and we hypothesize that they are stored in cytoplasmic granules. We analyzed the cellular localization of the *SKS14* and *AT59* Arabidopsis mRNAs, which are orthologues of the tobacco *NTP303* and tomato *LAT59* pollen mRNAs, respectively, by artificially labeling the transcripts with a MS2‐GFP chimera. A MATLAB‐automated image analysis helped to identify the presence of cytoplasmic *SKS14* and *AT59* mRNA granules in mature pollen grains. These mRNA granules partially colocalized with VCS and DCP1, two processing body (PB) proteins. Finally, we found a temporal correlation between SKS14 protein accumulation and the disappearance of *SKS14* mRNA granules during pollen germination. These results contribute to unveil a mechanism for translational regulation in *Arabidopsis thaliana* pollen.

## 1 INTRODUCTION

Arabidopsis pollen development involves two stages: an early stage that includes microspores and bicellular pollen followed by a late stage of tricellular and mature pollen. These two stages differ in their transcriptional profiles: early genes expressed during the microspore stage decrease their abundance before pollen maturation. In turn, late genes are expressed after asymmetric mitosis and accumulate during pollen maturation, bringing about a stable pool of mRNAs that govern pollen germination and pollen tube early growth. Thereby, at anthesis, all proteins or mRNAs required for germination and pollen tube growth would be present (Boavida, Becker, & Feijó, 2005). Consistent with this, in many species, pollen germination is independent of transcription but dependent on translation (Twell, 1994).

Pollen tube growth is a process that occurs in an explosive way, and tip extension requires a rapid increase in pectin amount. Some of the late pollen genes encode proteins homologous to enzymes linked to pectin metabolism, including polygalacturonases (Brown & Crouch, 1990; Niogret, Dubald, Mandaron, & Mache, 1991; Rogers & Lonsdale, 1992), pectin methylesterases (Mu, Stains, & Kao, 1994; Wakeley, Rogers, Rozycka, Greenland, & Hussey, 1998), and pectate lyases (Rogers, Harvey, & Lonsdale, 1992; Wing et al., 1989). The tomato late gene *LAT59* codifies a protein related to the pectate lyase family, potentially involved in cell wall degradation by pectin cleavage. The translation of *LAT59* mRNA is highly regulated and occurs since final stages of pollen development (Curie & McCormick, 1997). In turn, the tomato *LAT52* is a pollen gene that codifies a cysteine‐rich extracellular protein involved in pollen hydration and pollen germination (Muschietti, Dircks, Vancanneyt, & McCormick, 1994). *LAT52* transcript levels gradually increase during pollen development reaching its maximum at pollen maturity (Twell, Klein, Fromm, & McCormick, 1989).

Another example is the tobacco *NTP303* gene, which is transcribed through pollen development from the early binucleate stages (Weterings et al., 1992) and translated once germination occurs (Wittink et al., 2000). NTP303 has homology with ascorbate oxidases, and according to its time of expression, it would be linked to pollination or fertilization (Schrauwen et al., 1999).

Processing body (PB) are highly conserved cytoplasmic organelles involved in translation inhibition, mRNA degradation, and storage. PB formation includes translationally repressed messenger ribonucleoproteins (mRNPs) that aggregate into larger structures through protein–protein interactions. mRNPs localized in PBs can be degraded or undergo a rearrangement where translation initiation factors are recruited, thus allowing mRNAs to reenter polysomes (Decker & Parker, 2012).

As in yeast and animals, it has been shown that *Arabidopsis thaliana* PBs include decapping factors and coactivators, such as decapping protein one (DCP1), decapping protein two (DCP2), decapping protein five (DCP5), and varicose (VCS) (Xu & Chua, 2009; Xu, Yang, Niu, & Chua, 2006). The knockout of these genes affects the growth of vascular and epidermal cells, stomata, and root hairs, suggesting that decapping and/or PBs have a fundamental role during plant development (Maldonado‐Bonilla, 2014; Xu et al., 2006).

Here, we focus in the Arabidopsis mature pollen *SKS14* and *AT59* mRNAs (Loraine, McCormick, Estrada, Patel, & Qin, 2013) which are putative orthologues of the tobacco *NTP303* and tomato *LAT59* mRNAs, respectively. We found *SKS14* and *AT59* mRNAs in cytoplasmic granules that colocalize with the PB markers VCS and DCP1. Finally, we show that SKS14 protein accumulates during pollen germination while the number of *SKS14* mRNA granules decreases. These observations are compatible with the notion that the *SKS14* mRNA is released from PBs to allow translation in a controlled manner.

## 2 MATERIAL AND METHODS

### 2.1 Plant material and growth conditions

Sterilized seeds from *Arabidopsis thaliana* (ecotype Columbia‐0) wild‐type, single mutants, and transgenic plants were plated on 0.5X Murashige and Skoog (1962) medium with 1% sucrose and selective agent (50 mg/l kanamycin) if necessary and cold stratified 4 days in dark at 4°C. Seeds were germinated and grown under continuous light at 22°C for 7 days. Seedlings were then transferred to soil or peat, mixed with vermiculite and perlite (2:1:1), and grown in a chamber at 22°C under long‐day (16/8 hr light/dark) photoperiod and 60% relative humidity.

### 2.2 Plasmid constructs

MS2 system is based on the strong association between bacteriophage MS2 capsid protein (MCP) and six repeat loops of a 19‐nucleotide fragment containing bacteriophage's replicase start codon (Bertrand et al., 1998). The *pMS2‐GFP* and *pSL‐MS2‐12X* plasmids were donated by Robert Singer (Addgene plasmid #27121 and #27119, respectively) (Bertrand et al., 1998; Fusco et al., 2003).

*MS2* control vector was generated through an LR recombination system (Invitrogen) using the binary vector pK7WG2D. After PCR amplification from the *pMS2‐GFP* plasmid, the *GFP‐MCP* fragment was inserted into the pZD05 vector under the control of LAT52 promoter, obtaining the *pLAT52::GFP‐MCP*. Then, the *pLAT52::GFP‐MCP* fragment was inserted in the pENTR1a entry vector and then recombined in the binary vector pK7WG2D. All plasmids were confirmed by sequencing.

*SKS14* and *AT59* 5′UTRs and coding regions were cloned by PCR from Arabidopsis mature pollen cDNA in the pSL‐MS2‐12X plasmid. The *SKS14‐MS2‐12X* and *AT59‐MS2‐12X* fragments were PCR‐cloned into pZD05. *pLAT52::SKS14‐MS2‐12X* and *pLAT52::AT59‐MS2‐12X* fragments were PCR‐cloned and inserted into the binary vector *pK7WG2D‐pLAT52::GFP‐MCP* obtaining *pLAT52::GFP‐MCP*/*pLAT52::SKS14‐MS2‐12X* and *pLAT52::GFP‐MCP*/*pLAT52::AT59‐MS2‐12X*. All plasmids were confirmed by sequencing.

*pLAT52::RFP‐VCS* and *pLAT52::RFP‐DCP1* vectors were generated using the binary vector pK7WG2D. The VCS or DCP1 fragments were amplified from the pda09249 DNA stock (ABRC) or Arabidopsis mature pollen cDNA, respectively. They were inserted in the pENTR1a entry vector and then recombined in pK7WG2D. The *pLAT52‐RFP* fragment was amplified from the pZD05 vector, digested, and cloned in pK7WG2D containing VCS or DCP1. All plasmids were confirmed by sequencing.

*pSKS14::SKS14‐RFP* vector was generated using binary vector pGWB408. The *SKS14* coding region and the *RFP* fragment were amplified from Arabidopsis cDNA and from the pZD05 vector, respectively, and cloned in the pENTR1a vector. The 35S promoter of the pGWB408 vector was replaced by a 1091‐bp fragment corresponding to the SKS14 promoter that was cloned by PCR from Arabidopsis cDNA. All plasmids were confirmed by sequencing.

EHA105 Agrobacterium strain was used for transformation. Plant transformation was carried on by floral dip method (Clough & Bent, 1998).

### 2.3 Microscopy analysis

Mature pollen grains of twenty‐day‐old plants were collected and stored in Tris–EDTA buffer in the presence or absence of puromycin (100 μg/ml) for 1 hr at 22°C. Images were taken with a fluorescence microscope Olympus BX41 or in a confocal microscope Olympus IX81 FV1000 (Laser 488 nm, filter BP 505‐525, or Laser 543 nm, filter BP 560‐620) and analyzed by MATLAB scripts.

### 2.4 MATLAB scripts

#### 2.4.1 mRNA granules detection

In order to analyze the cytoplasmic granules by MATLAB (Fig. S2), initially a mask was applied to eliminate vegetative nucleus and facilitate the cytoplasm visualization. Then, the image was split into 49 boxes and the average fluorescence of each box was determined (Fig. S2C). Those groups of pixels that were between 5 and 30 pixels in size and had an average fluorescence higher than three standard deviations from the average fluorescence of the corresponding box were marked with a yellow point. The grid was moved 20 pixels to the right and down, and a new round of analysis was carried out. Those groups of pixels that followed similar parameters were marked with a light blue circle (Fig. S2D). Pixels detected with both analysis (yellow point and light blue circle) were processed in a new round of the script to confirm the cytoplasmic granules (Fig. S2E). The validated cytoplasmic granules were marked with a red point and a green circle, indicating the center and the approximate area of the granule, respectively (Fig. S2F). Determination of the number of cytoplasmic granules was carried out by taking one image of the central plane of each pollen grain; by this approach, we obtained an approximated value of the number of cytoplasmic granules for each pollen grain analyzed. All the analysis was carried out in the presence of puromycin 50 μg/ml.

#### 2.4.2 PBs detection

A similar MATLAB script used for the mRNA granules was applied for PBs detection except that positive PB marker protein foci were defined between 5 and 80 pixels.

#### 2.4.3 Colocalization analysis

To address statistical significance of the proximity between the mRNAs granules and PB marker protein foci (i.e., whether or not they may colocalize by chance given the specific layout of mRNAs and PBs within an image), a shuffling analysis was performed using a custom‐made MATLAB routine. After confirming the cytoplasmic granules and the PB marker protein foci, both were modeled as circles in the 2D plane using the center of mass and area obtained from previously described analysis. The random distribution of the distance between the mRNA granule and the nearest PB foci was obtained by randomly repositioning the mRNA granule 10,000 times while keeping the PB's foci layout fixed. From that random distribution, we proceeded to obtain the *p*‐value for the experimentally obtained distance. The sum of the radii of the mRNA granule and the nearest PB marker protein foci was used to determine whether they were colocalizing, proximal, or distant. All the analyses were carried out in the presence of puromycin 50 μg/ml.

### 2.5 Statistical analysis

Results are expressed as scattered plots. Points represent data dispersion for *n* = 7‐10 quantifications of cytoplasmic granules per pollen grain (each n corresponds to the media of the number of cytoplasmic granules of 10‐20 pollen grains) and *n* = 10‐25 for determination of fluorescence intensity (each *n* corresponds to one vegetative nucleus fluorescence intensity measurement); probability values of <.1 were considered statistically significant. Statistical analysis of the data and further processing were performed using GraphPad Prism version 5.00 for Windows (GraphPad Software).

## 3 RESULTS

### 3.1 *SKS14* and *AT59* mRNAs form cytoplasmic granules in mature pollen

LAT59 protein is present at final stages of pollen development and its amount increases after pollen germination (Curie & McCormick, 1997) while NTP303 is synthesized only once pollen germinates (Wittink et al., 2000). We sought to investigate the post‐transcriptional regulation of the Arabidopsis *LAT59* and *NTP303* orthologues. Arabidopsis *AT59* (At1 g14420) is the *LAT59* ortholog (Kulikauskas & McCormick, 1997). Among the putative NTP303 orthologues, we focused on the SKU5 similar (SKS) family that has 19 members in Arabidopsis (Sedbrook et al., 2002). Among them, *SKS11*,* SKS12*,* SKS13,* and *SKS14* are expressed in pollen (Honys & Twell, 2000). We then considered the length and free energies of their 5′UTRs regions and compared these parameters to those of NTP303 5′UTR. Furthermore, SKS14 is the pollen SKS gene that shows the highest increment in expression levels as pollen development proceeds, showing a maximum in mature pollen (Honys & Twell, 2000). This makes SKS14 a good candidate to test our hypothesis and therefore was chosen for this study.

**Figure 1:**
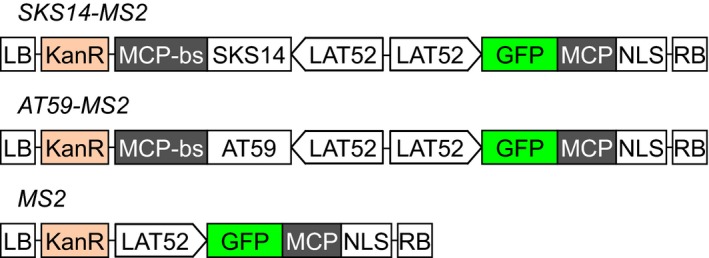
mRNA detection by the MS2 system. The two depicted constructs were inserted in the same vector: GFP fused to the MS2 coat protein (MCP) with a nuclear localization signal (NLS), and the *SKS14* or *AT59* transcripts fused to MCP‐binding site (MCP‐bs). A control construct termed *MS2* encodes the GFP‐MCP chimera and no target mRNA. The three constructs *SKS14‐MS2*,*AT59‐MS2,* and *MS2* were under the control of the pollen‐specific promoter LAT52. LB and RB, left and right borders of the translocation cassette, respectively

**Figure 2:**
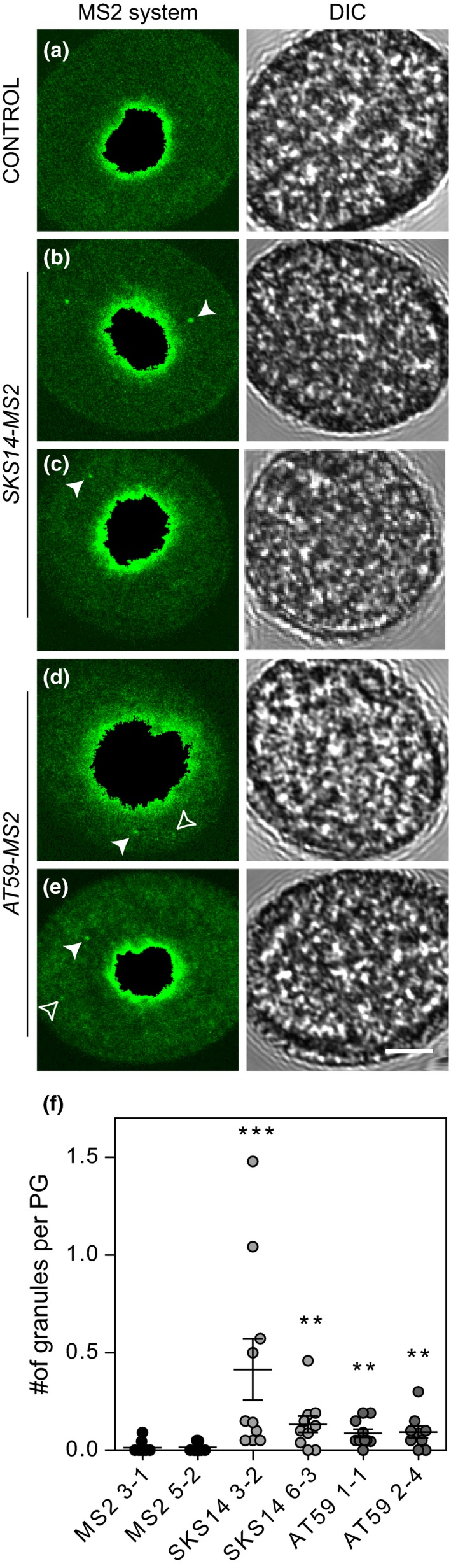
Arabidopsis *SKS14* and *AT59* mRNAs form cytoplasmic granules in mature pollen grains. (a) Confocal images of a representative pollen grain from the control *MS2* line. A mask was applied to eliminate vegetative nucleus and facilitate cytoplasm visualization. (b‐e) Representative images of two independent *SKS14‐MS2* (b and c) and two *AT59‐MS2* lines (d and e). In the left panels, white arrowheads show examples of cytoplasmic granules identified by the MATLAB script while empty arrowheads show cytoplasmic aggregates that were not detected by the MATLAB script. Right panels, DIC images. Size bar, 5 μm. (f) Quantification of cytoplasmic granules. Each point corresponds to the mean value of an independent sample that included 20 pollen grains. The media and standard error for each transgenic line are shown. Statistical significance (Mann–Whitney test) relative to the control line MS2 3‐1 is indicated (***p < .001 and **p < .01)

Paralleling the fate of maternal mRNA in animal oocytes, we hypothesize that *SKS14* and *AT59* mRNAs are stored in cytoplasmic granules during pollen development. To analyze the subcellular localization of these transcripts in vivo, we artificially labeled the mRNA using the MS2‐MCP system, which is based on the strong binding of the bacteriophage MS2 coat protein (MCP) to specific RNA loops termed MCP‐binding site (Lampasona & Czaplinski, 2016). The GFP‐MCP protein was fused to a nuclear localization signal (NLS) so that only the GFP‐MCP bound to the target mRNAs is found in the cytoplasm. Fig. S1 shows that the free GFP‐MCP protein is largely accumulated in the vegetative nuclei identified by DAPI staining. We obtained independent Arabidopsis transgenic lines containing the GFP‐MCP construct together with either the *SKS14* or the *AT59* mRNA fused to the MCP‐binding site, termed *SKS14‐MS2* and *AT59‐MS2*, respectively (Fig. 1). All constructs are under the control of the tomato pollen‐specific promoter LAT52 (Twell, Yamaguchi, Wing, Ushiba, & McCormick, 1991). For comparison, we generated control transgenic plants termed *MS2* lines carrying only the GFP‐MCP construct (Fig. 1). With these tools, we found that both *SKS14* and *AT59* messengers form granules in the cytoplasm of mature pollen grains (Fig. 2; Table 1).

**Table 1:** Quantification of cytoplasmic granules per pollen grain in WT pollen. Mean values from 20 pollen grains in independent samples are shown, and the media and standard error for each transgenic line are indicated. *N*: number of pollen grains analyzed. ND: Not determined (Mann–Whitney test)

Line	Media	Standard error	*N*	*p*‐value	*p*‐value summary
*MS2 3‐1*	0.014	0.010	10	ND	ND
*SKS14 3‐2*	0.413	0.156	10	.0003	***
*SKS14 6‐3*	0.133	0.042	10	.0053	**
*AT59 1‐1*	0.087	0.021	10	.0029	**
*AT59 2‐4*	0.093	0.031	9	.0092	**

Representative images of pollen grains showing cytoplasmic granules of *SKS14‐MS2* mRNA and *AT59‐MS2* mRNA are depicted in Fig. 2b‐e. A representative control *MS2* pollen grain is shown in Fig. 2a.

To perform an automated unbiased analysis of these images, we implemented a MATLAB script that detects granules in the cytoplasm, applying a mask to the nucleus and measuring pixel size and fluorescence intensity (see Material and Methods and Fig. S2). Fig. 2f shows that the number of cytoplasmic granules of fluorescent protein in the *SKS14‐MS2* and *AT59‐MS2* lines was 10‐40 times higher than in the control *MS2* line (Table 1).

The number of mRNA granules per pollen grain is somehow lower than expected considering that the strong LAT52 promoter was used. Given that a whole scan of the entire pollen grain along *Z‐*axis rapidly quenched the fluorescent GFP signal, a maximum of five confocal sections of each pollen grain were analyzed, with a final size of 2.5 um thick. This represents about 10% of the volume of an Arabidopsis pollen grain, and thus, we speculate that the total number of RNA granules would be 10 times larger than the experimental value. In addition, the presence submicroscopic RNA granules of less than the resolution of the confocal microscope (~150 nm) should also be considered. A second factor that may be introducing a systematic error is that granules close to the perinuclear region were not included, due to the strong GFP nuclear signal and because most of the potential positives close to the perinuclear region were extensions of the vegetative cell nuclei indentations. Thus, a significant proportion of mRNA granules was lost in our analysis, but at the same time, we largely reduced the chances of including false positives.

To test that the differences observed in the number of granules were not due to dissimilar expression levels of the GFP‐MCP chimera in the different Arabidopsis lines, we measured the fluorescence intensity in the vegetative nuclei, where nonbound GFP‐MCP accumulates due to the NLS included in the construct. Although GFP‐MCP intensity varies between lines, we found that there is no correlation between the nuclear fluorescence intensity and the number of cytoplasmic granules, strongly supporting that these granules are specific and are not due to the overexpression of heterologous GFP‐MCP protein (Fig. S3).

### 3.2 *SKS14* and *AT59* mRNA cytoplasmic granules colocalize with PB proteins

**Figure 3:**
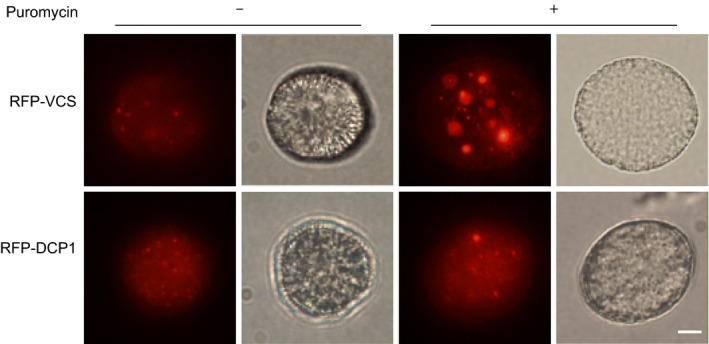
The PB proteins VCS and DCP1 form cytoplasmic foci in mature pollen. Representative images of mature pollen grains in the absence (‐) or presence (+) of puromycin 50 μg/ml. DIC images are shown in the right panels. Size bar, 5 μm

After demonstrating that the *SKS14* and *AT59* mRNAs accumulate in cytoplasmic granules in mature pollen, we sought to determine whether these granules contain PBs marker proteins. We first investigated the localization of VCS and DCP1 in mature pollen by expressing *RFP‐VCS* and *RFP‐DCP1* under the LAT52 promoter. Fig. 3 shows that *RFP‐VCS* and *RFP‐DCP1* appeared as cytoplasmic foci. As expected, the size of these bodies increased upon incubation with puromycin, a drug that inhibits translation elongation‐releasing mRNAs from ribosomes, thus allowing mRNA recruitment to PBs (Thomas, Loschi, Desbats, & Boccaccio, 2011). The effect was stronger for the *RFP‐VCS* construct where PBs increased both in size and number upon exposure to puromycin (Fig. 3).

**Figure 4:**
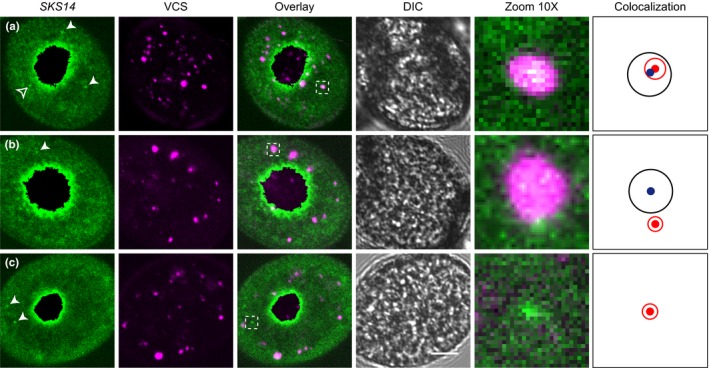
Colocalization of *SKS14* mRNA with *RFP‐VCS*. (a) Confocal image of a representative mature pollen grain showing high colocalization between *SKS14* mRNA and a RFP‐VCS body. (b) Confocal image of a representative mature pollen grain showing a *SKS14* mRNA cytoplasmic granule contiguous to a RFP‐VCS body. (c) Confocal image of a representative cytoplasmic granule with no relationship with any RFP‐VCS body. In the left panels, white arrowheads show examples of cytoplasmic granules identified by MATLAB while empty arrowheads show cytoplasmic aggregates not detected by MATLAB. The insets in the merged (“Overlay”) column are enlarged on the 10X panels. In the “Colocalization” column, the blue point and black circle indicate the localization and size of the VCS body, respectively, and the red point and circle correspond to the *SKS14* mRNA granule. DIC images are shown. Size bar, 5 μm

Next, we generated four different Arabidopsis lines by crossing the *SKS14‐MS2* or *AT59‐MS2* with either the *RFP‐VCS* or *RFP‐DCP1* lines. Automated PBs and mRNA granule detection was performed by a MATLAB script as above (for details see “Materials and Methods”). To investigate colocalization, the two structures were modeled as circles using the area and center obtained with the MATLAB algorithm, as detailed in Materials and Methods. Colocalization was considered to occur when the distance between the centers of the structures was equal or lower than the sum of their radii. Proximity was defined when the distance was larger but less than twice the sum of their radii. Finally, no relationship was assumed if the mRNA granule and the PB were separated by more than twice the sum of their radii (Fig. S4). The position and size of the mRNA granules and PBs were determined by specific MATLAB scripts (for details see “Materials and Methods”), merged the results and determined whether the mRNA granules colocalized, were proximal, or showed no relationship to the PBs protein markers. Fig. 4 a, b, and c shows representative *SKS14‐MS2 RFP‐VCS* pollen grains depicting the three types of spatial colocalization relationships. We performed the same study for three additional lines, *SKS14‐MS2 RFP‐DCP1* (Fig. S5), *AT59‐MS2 RFP‐VCS* (Fig. S6), and *AT59‐MS2 RFP‐DCP1* (Fig. S7), with similar results.

To address the extent of random colocalization in these images, we performed a shuffling analysis (for description of the script, see “Materials and Methods”). Fig. S4 shows examples of shuffling analysis for each one of the colocalization groups. At an alpha value of 0.05, the experimental distance observed for colocalized and proximal granule pairs, but not for distant granules, was significantly different than the values expected by chance.

Then, we quantified the number of mRNA granules that colocalized or were nearby PBs (Table 2). We found that around 20% of the *SKS14* mRNA bodies colocalized with either VCS or DCP1, while approximately 10% of them were found proximal. Similarly, 10% of the *AT59* mRNA granules colocalized with the VCS or DCP1 bodies and a similar fraction was found nearby them (Table 2). These results suggest that both *SKS14* and *AT59* mRNAs showed partial localization with PBs identified by VCS or DCP1.

**Table 2:** *SKS14* and *AT59* mRNA cytoplasmic granules colocalize with PB proteins. Percentages of granules showing colocalization or proximity for each mRNA and PB protein pair are indicated. *N*: number of pollen grains analyzed

mRNA/protein	Colocalization (%)	Proximal (%)	*N*
*SKS14*‐VCS	19.6	6.5	46
	21.4	16.7	42
*SKS14*‐DCP1	23.8	0	21
	20	10	30
*AT59*‐VCS	13	9.5	63
	14.3	11.4	35
*AT59*‐DCP1	11.4	11.4	35
	8.8	0	34

### 3.3 SKS14‐RFP protein is expressed at early stages during pollen germination

**Figure 5:**
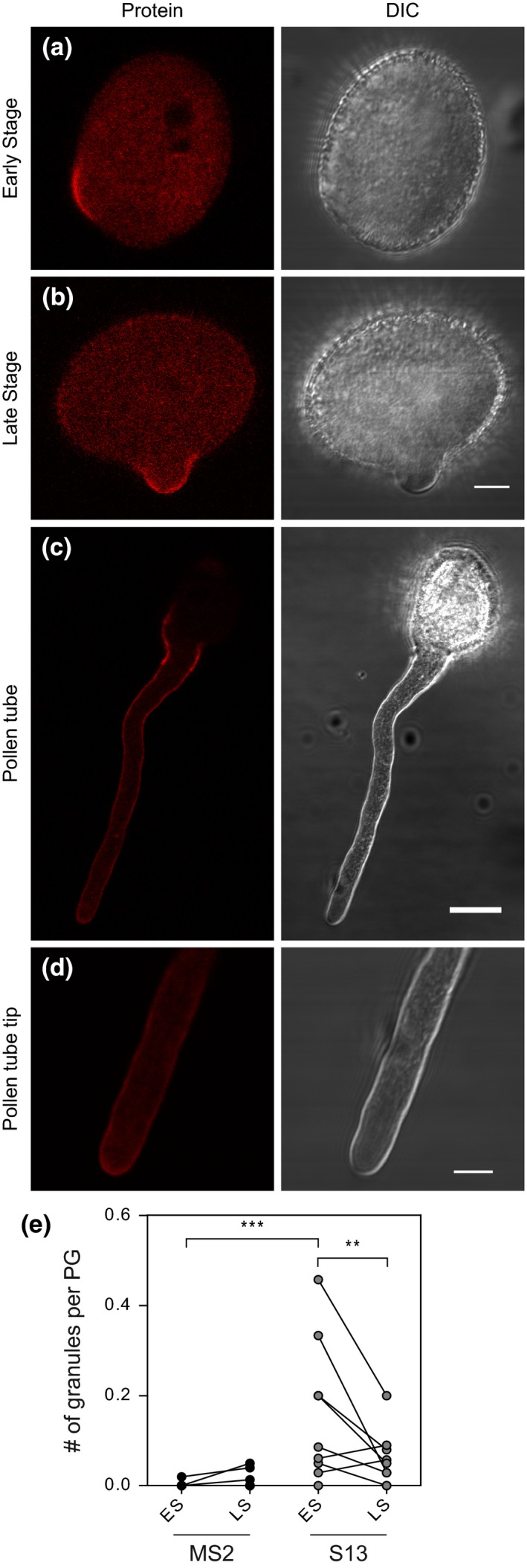
SKS14‐RFP protein is expressed from early stages of germination and localizes at the margins of pollen tubes. Representative images of S2 line pollen grains expressing SKS14‐RFP protein (left panels). Right panels, DIC images. Size bars, 5 μm for early and late stages (a and b) and 15 μm for the pollen tube (c). (d) A 5X magnification shows localization at the tip (Size bar, 5 μm). (e) Quantification of *SKS14* mRNA cytoplasmic granules per pollen grain compared to a MS2 control (MS2 3‐1). ES and LS, early and late stages of pollen germination, respectively. Each point represents the mean value of independent samples including 9‐10 pollen grains. Lines link data from paired samples (same experiment). Statistically different values are indicated (***p < .001 and **p < .01). The *p*‐value for the ES‐MS2/LS‐MS2 pair was 0.99, and for the LS‐MS2/LS‐S13 pair, 0.48 (two‐way ANOVA randomized block, Bonferroni post‐test)

Next, we asked when the *SKS14* mRNA is translated. We generated two independent Arabidopsis lines (S2 and S13) that express both pLAT52::*GFP‐MCP*/pLAT52::*SKS14‐MS2* and pSKS14::*SKS14‐RFP* simultaneously. Fig. 5 shows that the SKS14‐RFP protein started to be detected during pollen germination, being localized to the region where the pollen tube will emerge (Fig. 5a‐b). Later, SKS14‐RFP is accumulated at the margins of pollen tubes as long as they grow (Fig. 5c‐d), suggesting that there is an increase in the total amount of SKS14‐RFP. These observations agree with previous results that show that the tobacco NTP303 is present at the cell wall and at callose plugs of growing pollen tubes (Wittink et al., 2000).

In general, mRNA translation correlates with silencing foci remodeling or dissolution (reviewed in Thomas et al., 2011; Pascual, Maschi, Luchelli, & Boccaccio, 2014). We wonder whether *SKS14* expression involves changes in the number of *SKS14* mRNA granules. We analyzed in the double‐transgenic lines (S2 and S13) the presence of *SKS14* mRNA granules at the early (ES) and late (LS) stages of pollen germination (Fig. 5a‐b). We found a statistically significant decrease in the number of granules at the late stage of pollen germination when compared to the corresponding early stage in the S13 line (Fig. 5e). We also found for both S2 and S13 lines that the number of fluorescent mRNA granules at the early stage, but not at the late stage, was significantly higher in comparison with the control *MS2* line (ES‐MS2/ES‐S2 pair *p* = .019 (*p < .1) and LS‐MS2/LS‐S2 pair *p* = .25; ES‐MS2/ES‐S13 pair *p* = .0002 (***p < .001) and LS‐MS2/LS‐S13 pair *p* = .48). These results suggest that during pollen germination, when the SKS14‐RFP protein is being synthetized, there is a simultaneous decrease in the amount of *SKS14* mRNA granules, suggesting that the *SKS14* mRNA granules release their content to allow a controlled production of the SKS14 protein.

## 4 DISCUSSION

Both in plants and animals, gamete development depends on the translation of stored mRNAs. The post‐transcriptional control of genes expressed during the spermatogenesis in fly and mouse is well known (Lasko, 2012; Nguyen‐Chi & Morello, 2011). In plants, the translational inhibitor cycloheximide, but not the transcriptional inhibitor actinomycin D, inhibits early pollen tube growth, suggesting that translation of preexisting mRNAs is required (Hao, Li, Hu, & Lin, 2005). Studies on the regulation of the tobacco *NTP303* and tomato *LAT59* genes demonstrated that they are both transcribed during pollen development while their proteins are mostly or exclusively synthesized after germination, respectively (Curie & McCormick, 1997; Hulzink et al., 2002). Antisense NTP303 plants are male sterile due to the arrest of pollen tubes within the style (de Groot et al., 2004). Fig. 5 shows a negative correlation between the presence of *SKS14* mRNA granules and RFP‐SKS14 expression, which is compatible with a role for the granules in mRNA storage. We found that upon initiation of pollen germination, the related SKS14 protein localizes where the pollen tube emerges, and later in the pollen tube margins. Tobacco NTP303 is related to ascorbate oxidases and localizes at the plasma membrane (Wittink et al., 2000), and we propose that SKS14 could have a similar role in Arabidopsis pollen.

Here, we visualized the mRNAs of the Arabidopsis *SKS14* and *AT59* mRNAs in mature pollen and during germination. We identified *SKS14* and *AT59* mRNA in cytoplasmic granules related to PBs in mature pollen and propose that these bodies would function in mRNA storage while waiting for being translated. In addition, the presence of PB components in these RNA granules may be linked to mRNA degradation. However, we favor the notion of mRNA storage given that SKS‐RFP accumulates latter during pollen tube growth (Fig. 5c).

It is generally accepted that mRNAs translationally inactive are stored in stress granules (SGs), processing body (PB), or related organelles (Decker & Parker, 2012; Thomas et al., 2014). We propose that an active exchange of mRNAs between PBs and polysomal mRNPs finely tune gene expression during the late stages of pollen development and during germination. Supporting this, in a pioneer work, the *NTP303* mRNA was found in polysomal and in a fraction of particles resistant to EDTA/puromycin treatment referred to as EPPs (Honys, Combe, Twell, & Capková, 2009). These particles have been proposed as long‐term storage complexes. EPPs include a set of mRNAs that are stored and translationally repressed at early stages of pollen development. Some of the stored mRNAs are massively translated at late stages of pollen development and/or transported to the pollen tube where they are translated. Relevantly, most of the translationally inactive *NTP303* mRNA was present both in the polysomal and EPP fractions. Whether the EPPs and the granules identified here represent the same entity remains open. Against this possibility, VCS and DCP1 are absent from tobacco EPPs (Honys et al., 2003).

We also described the presence of processing body in Arabidopsis mature pollen. As in other cell types and organisms, RFP‐VCS and RFP‐DCP1 localized to discrete cytoplasmic bodies that become larger and more abundant upon treatment with puromycin, suggesting that pollen PBs recruit transcripts that are released from active polysomes. Accumulation of translationally repressed mRNAs in PBs has been previously reported in yeast, *Drosophila,* and mammals (Decker & Parker, 2012; Thomas et al., 2011). However, when analyzing the colocalization of *SKS14* and *AT59* mRNAs with VCS or DCP1, we found partial overlapping with about 30% of the granules in close contact. We speculate that pollen grains may contain several types of PBs, and only a fraction of them would contain VCS and DCP1. Several animal cell types display a variety of PB containing subsets of PB components, and in addition, it has been proposed that mRNAs are differentially located on PBs depending on their translational requirements (Weil et al., 2012). In mammalian neurons, only 50% of dendritic DCP1a bodies contain Hedls, the VCS ortholog, and vice versa, 50% of Hedls‐positive dendritic puncta contain DCP1a (Luchelli, Thomas, & Boccaccio, 2015). Likewise, in untreated mammalian cells, only 18% of the beta‐actin and Cerulean‐mini‐dystrophin mRNAs colocalized with PB using Hedls as a protein marker (Aizer et al., 2014). Further analysis including super‐resolution microscopy and the study of additional PB proteins and RNA regulation pathways will shed light on the dynamic relationship between pollen mRNAs and PBs and will allow a more complete understanding of their translational regulation.

In several examples, translation is repressed by proteins that bind to the UTRs and mRNA silencing is necessary for the proper localization and function of the encoded proteins (Chartrand et al., 2002; Jambor, Brunel, & Ephrussi, 2011). Several common elements in the 5′UTR of pollen‐expressed genes have been identified (Hulzink et al., 2003). Some of these consensus sequences are present in the *NTP303* 5′UTR and affect translation efficiency (Hulzink et al., 2002), thus opening the possibility that these pollen‐specific 5′UTR sequences play a regulatory role during development and germination. While the repression mechanism remains poorly understood, it has been proposed that translation of the *NTP303* mRNA is activated by factors that bind to the 5′UTR after pollen germination (Hulzink et al., 2002). In turn, binding of regulatory factors to the 5′UTR of the *LAT59* mRNA would inhibit translation at early stages and the release of the repressors upon pollen maturation would allow LAT59 protein synthesis (Curie & McCormick, 1997). Whether these 5′UTRs sequences mediate the targeting of these transcripts to PBs remain to be investigated. In this regard, it has been recently reported that CGG repeats in the 5′UTR of the Fragile *X* Mental Retardation 1 (*FMR1*) RNA mediate RNA localization into cytoplasmic granules (Rovozzo et al., 2016).

To securely define which pollen mRNAs are stored or translated, a robust and tight post‐transcriptional regulation is expected to occur. The presence of cytoplasmic granules that contain the *SKS14* and *AT59* mRNAs in mature pollen suggests a mechanism for translational regulation. Our results suggest that granules related to PBs store mRNAs to postpone their translation until necessary or that they regulate mRNA levels. An appealing speculation is whether the observations here reported for *SKS14* and *AT59* mRNAs can be extrapolated to other late pollen genes.

## AUTHOR CONTRIBUTIONS

MRS and JPM conceived the experiments; MRS, LS, SGT, GLB, LIP, and JPM designed the research; MRS performed the experiments; LS, SGT, and LIP provided technical assistance; MRS, LS, SGT, GLB, LIP, and JPM analyzed and interpreted the data; MRS and JPM wrote the manuscript with contributions of all the authors; GLB complemented the writing.
