# Supplementary material for: Two Arabidopsis late pollen transcripts are detected in cytoplasmic granules
Source: Plant Direct. 2017 Oct 16;1(4):e00012. doi: 10.1002/pld3.12 (PMC6508577; doi:10.1002/pld3.12)
Supplement: Supplementary file 1 [file PLD3-1-e00012-s001.pdf]

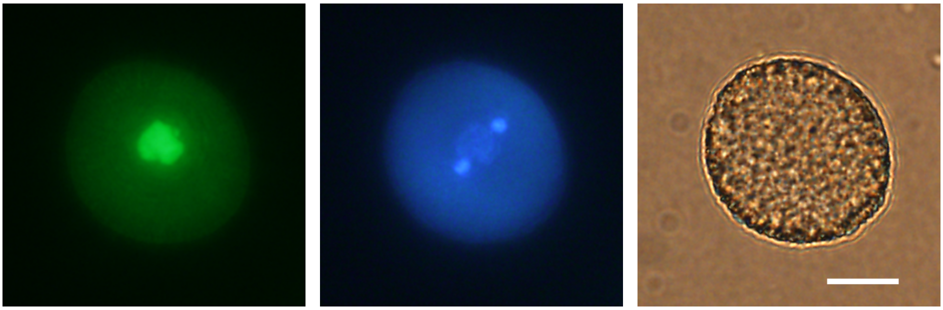

Figure S1. Free GFP-MCP is localized in the vegetative nuclei of mature pollen grains (left panel). Vegetative nuclei and sperm cells are stained by DAPI (middle panel) and bright field image is included (right panel). Size bar, 10 μm.

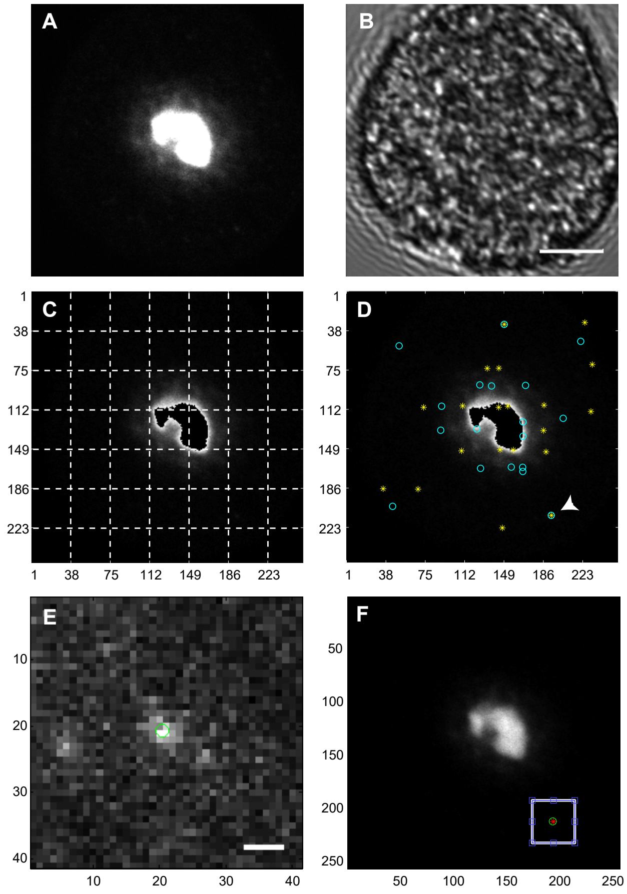

Figure S2. Detection of cytoplasmic granules by MATLAB script. Upper panel, representative pollen grain images of SKS14 mRNA with the saturated nucleus and a DIC image (**A** and **B**). Size bar, 5 µm. (**C**) The image was split in 49 boxes; the average fluorescence of each box was determined and a mask was applied to eliminate the vegetative nucleus and facilitate the cytoplasm visualization. (**D**) Those groups of pixels that are between 5 and 30 pixels in size and have an average fluorescence higher than three standard deviations from the average fluorescence were marked with a yellow point. The grid was moved 20 pixels to the right and down and a new round of analysis was carried out. Those groups of pixels that followed similar parameters were marked with a light blue circle. Arrowhead shows one group of pixels detected with both analysis (yellow point and light blue circle). (**E**) A box of 40 pixels side showing a granule detected by the MATLAB script. Size bar, 0.5 µm. (**F**) Original image of the analyzed pollen grain with the mark in red and green where the cytoplasmic granule was localized.

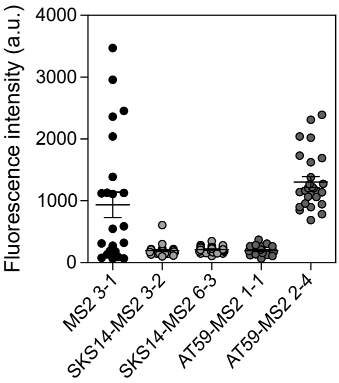

Figure S3. GFP-MCP expression levels. The fluorescence intensity was determined in the vegetative nucleus of mature pollen grains for the *MS2*, *SKS14-MS2* and *AT59-MS2* lines. Points represent data dispersion for n = 20-25. The media and standard error for each transgenic line is shown.

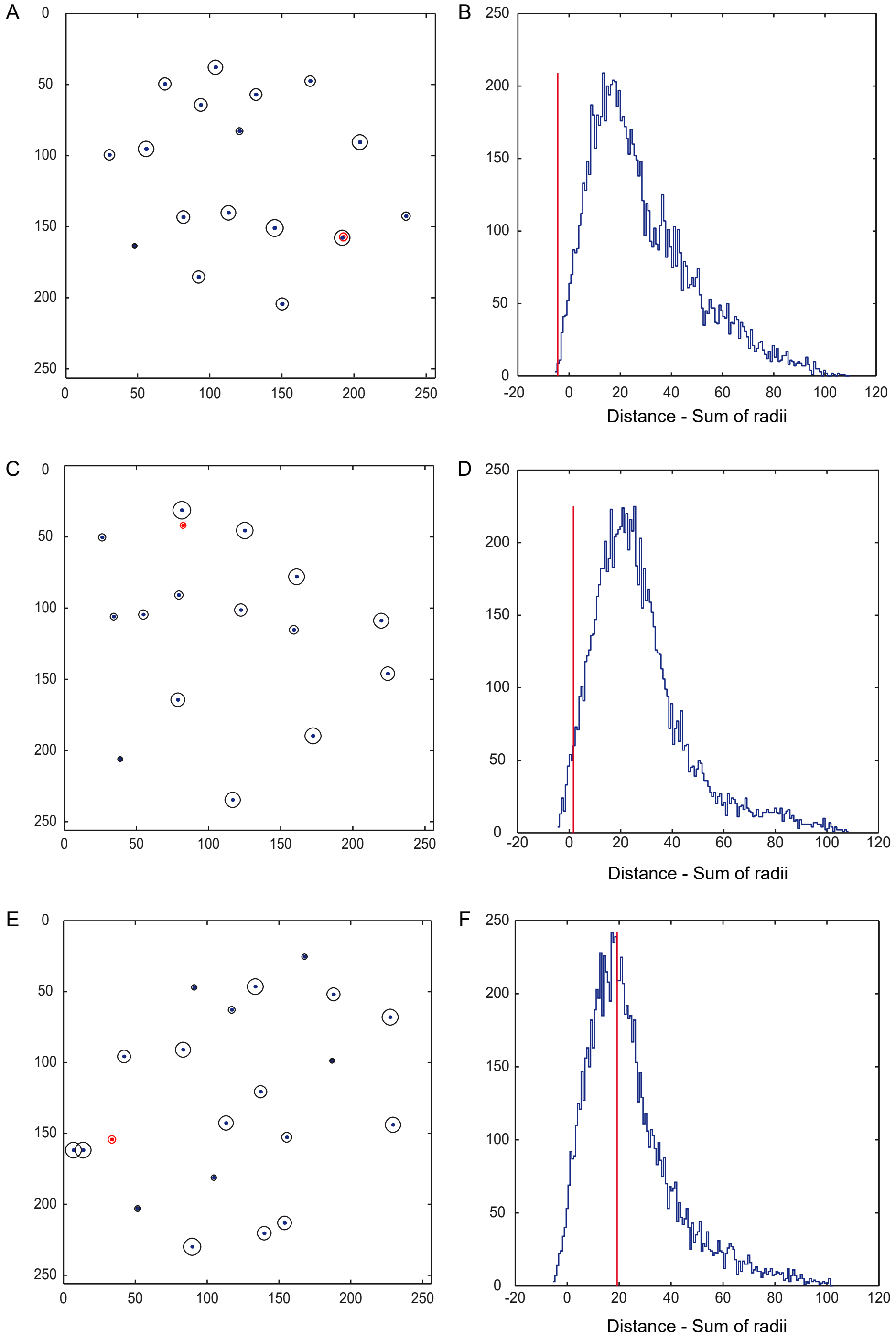

Figure S4. Statistical significance of the proximity between mRNAs granules and PBs using a shuffling analysis. Left panel, position and size of cytoplasmic granule (in red) and PBs marker protein foci (in blue). Right panel, histogram showing shuffling analysis for each group. The difference between the distance and the sum of the radii of the mRNA granule and the nearest PB was calculated, and the experimental and random values are depicted by blue and red lines respectively. (**A** and **B**) Colocalization: The difference between the distance and the sum of their radii is less than zero. p-value 0.0029. (**C** and **D**) Proximity: mRNA and protein distance is larger than the sum of their radii but lower than its double. p-value 0.0246. (**E** and **F**) No relationship. The mRNA and protein were separated by more than two times the sum of their radii. p-value 0.5229.

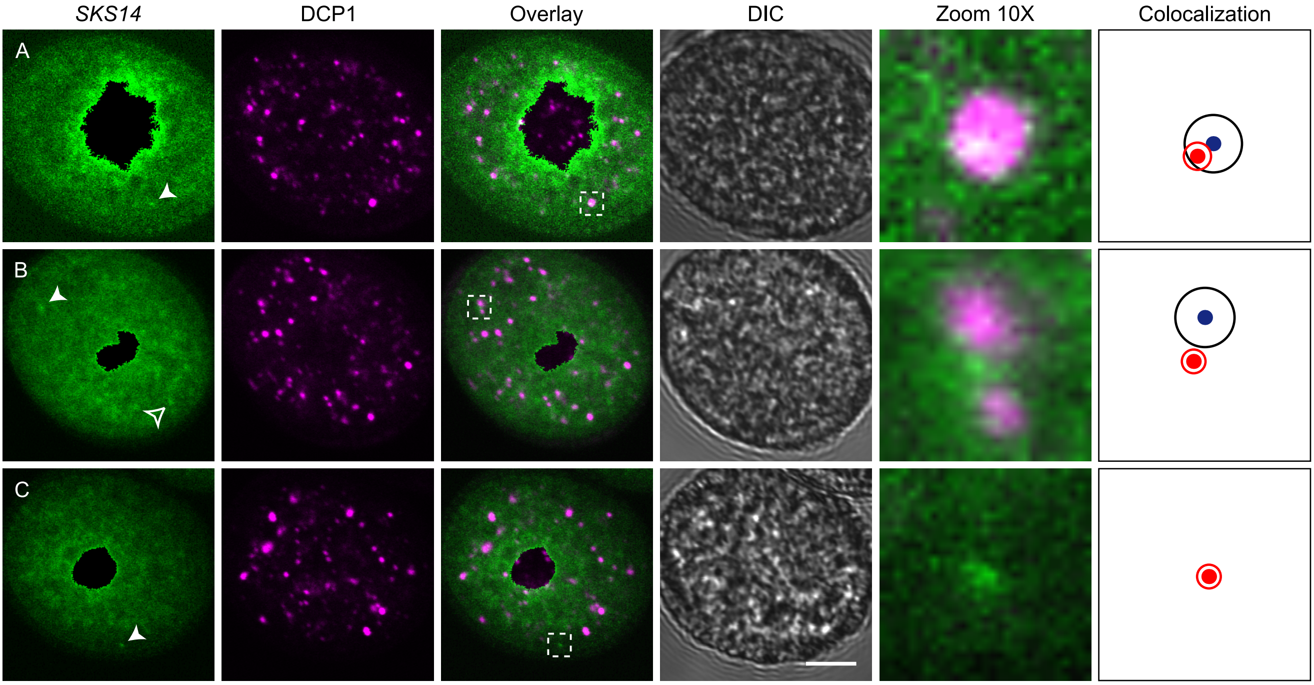

Figure S5. *SKS14* mRNA colocalized with DCP1. (**A**) Confocal image of a representative mature pollen grain showing high colocalization between *SKS14* mRNA and RFP-DCP1. (**B**) Confocal image of a representative mature pollen grain showing a *SKS14* mRNA cytoplasmic granule contiguous to a RFP-DCP1 focus. (**C**) Confocal image of a representative cytoplasmic granule distant from any RFP-DCP1 focus. In the left panels, white arrowheads show cytoplasmic granules confirmed by MATLAB while empty arrowheads show cytoplasmic aggregates not detected by MATLAB. The insets in the merged ("Overlay") column are enlarged on the 10X panels. In the "Colocalization" column, the blue point and black circle represent the localization and size of the DCP1 body; the red point and circle correspond to localization and size of the *SKS14* mRNA granule. DIC images are shown. Size bar, 5 µm.

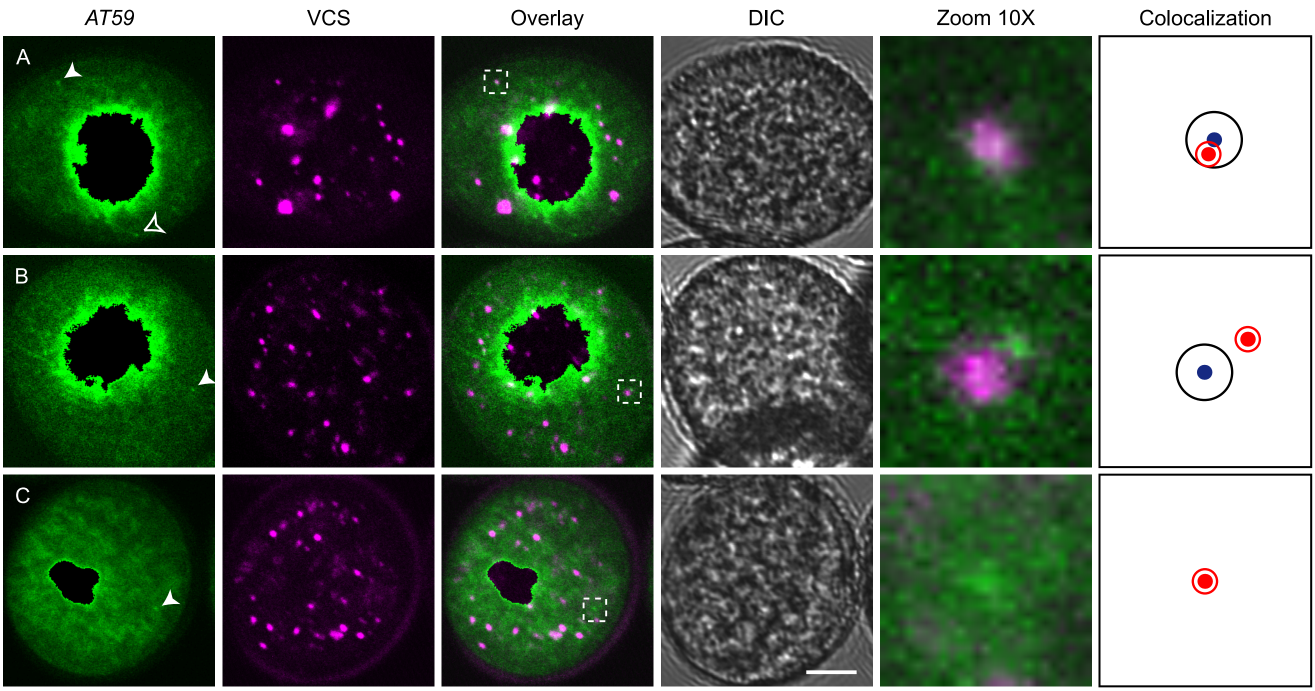

Figure S6. *AT59* mRNA colocalized with VCS. (**A**) Confocal image of a representative mature pollen grain showing high colocalization between *AT59* mRNA and RFP-VCS. (**B**) Confocal image of a representative mature pollen grain showing an *AT59* mRNA cytoplasmic granule contiguous to a RFP-VCS focus. (**C**) Confocal image of a representative cytoplasmic granule distant from any RFP-VCS focus. In the left panels, white arrowheads show cytoplasmic granules confirmed by MATLAB while empty arrowheads show cytoplasmic aggregates not detected by MATLAB. The insets in the merged ("Overlay") column are enlarged on the 10X panels. In the "Colocalization" column, the blue point and black circle indicates the localization and size of the VCS body; the red point and circle indicates the localization and size of the *AT59* mRNA granule. DIC images are depicted. Size bar, 5 μm.

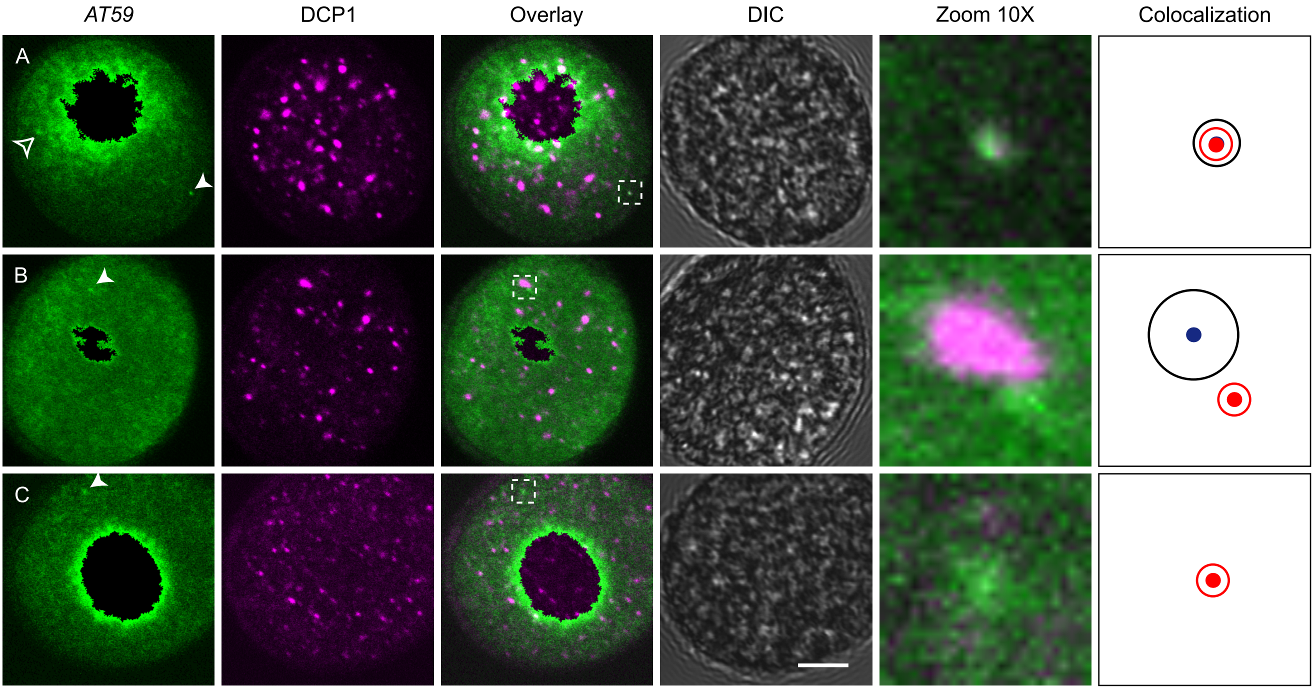

Figure S7. *AT59* mRNA colocalized with DCP1. (**A**). Confocal image of a representative mature pollen grain showing the colocalization between *AT59* mRNA and RFP-DCP1. (**B**) Confocal image of a representative mature pollen grain showing an *AT59* mRNA cytoplasmic granule contiguous to a RFP-DCP1 focus. (**C**) Confocal image of a representative cytoplasmic granule distant from any RFP-DCP1 foci. In the left panels, white arrowheads show cytoplasmic granules confirmed by MATLAB while empty arrowheads show cytoplasmic aggregates not detected by MATLAB. The insets in the merged ("Overlay") column are enlarged on the 10X panels. In the "Colocalization" column, the blue point and black circle indicates the localization and size of the DCP1 body; the red point and circle correspond to the localization and size of the *AT59* mRNA granule. DIC images are shown. Size bar, 5 μm.
