# Supplementary figures and images for: Two Arabidopsis late pollen transcripts are detected in cytoplasmic granules
Source: Plant Direct. 2017 Oct 16;1(4):e00012. doi: 10.1002/pld3.12 (PMC6508577; doi:10.1002/pld3.12)

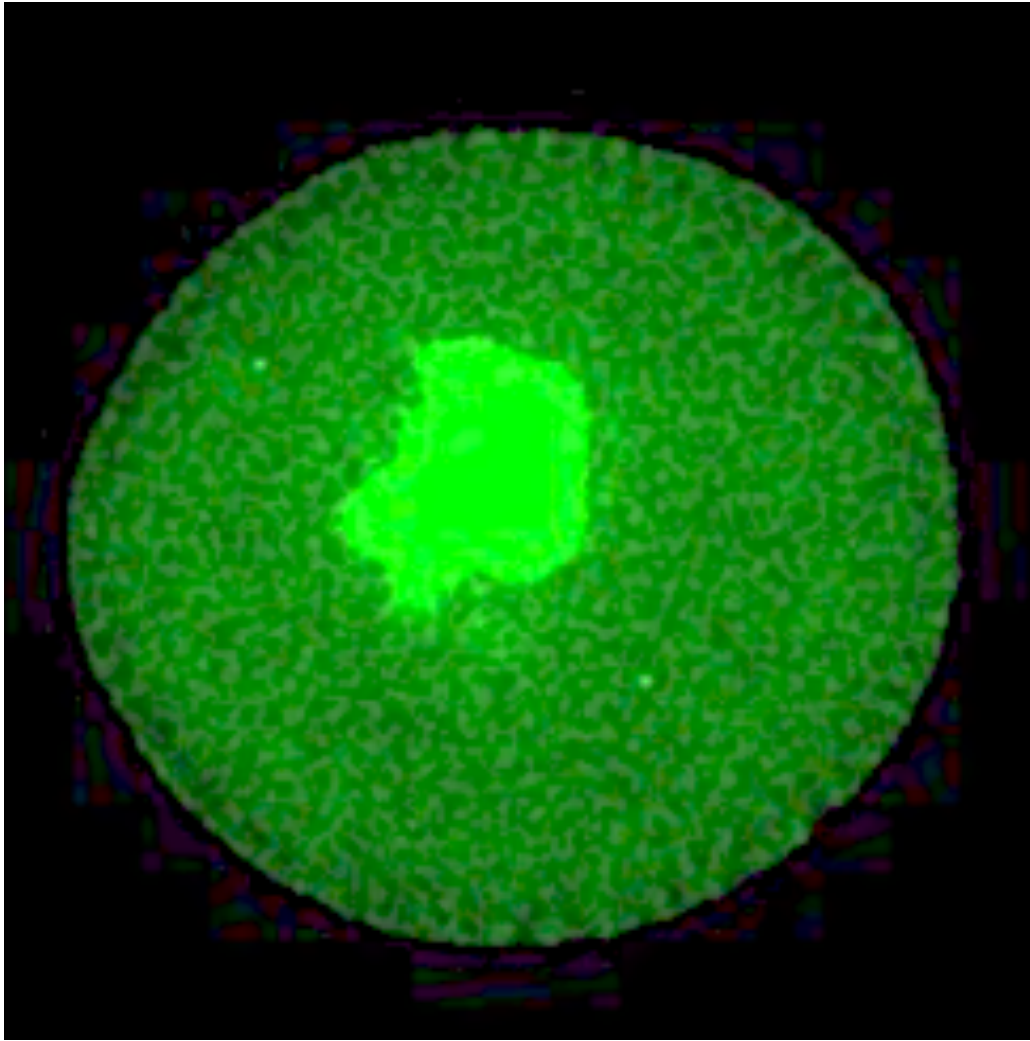

Supplement: Supplementary file 2 [file PLD3-1-e00012-s002.pdf]
